# Comparison of clinical and pathological findings of patients undergoing elective colectomy for uncomplicated diverticulitis

**DOI:** 10.1038/s41598-020-65727-1

**Published:** 2020-06-01

**Authors:** Antonio Pesce, Martina Barchitta, Antonella Agodi, Monica Salerno, Gaetano La Greca, Gaetano Magro, Saverio Latteri, Stefano Puleo

**Affiliations:** 1 Department of Medical and Surgical Sciences and Advanced Technologies “G.F. Ingrassia”, University of Catania, Via S. Sofia 78, 95123 Catania, Italy; 2 Department of Medical and Surgical Sciences and Advanced Technologies “G.F. Ingrassia”, Section of Legal Medicine, University of Catania, Via S. Sofia 78, 95123 Catania, Italy; 3 Department of Medical and Surgical Sciences and Advanced Technologies “G.F. Ingrassia”, Section of Anatomic Pathology, University of Catania, Via S. Sofia 78, 95123 Catania, Italy

**Keywords:** Gastroenterology, Colitis

## Abstract

Diverticular disease affects ∼5–10% people worldwide, yet the indications for elective colectomy in uncomplicated diverticulitis are unclear. As there is no strong scientific evidence regarding histology in diverticular disease, the primary outcome of the study was to analyze the degree of inflammation of colonic wall in patients that underwent elective colectomy for uncomplicated diverticulitis and to retrospectively assess the correlation between patient clinical history and pathological features of surgical specimens in order to find some predictive factors that may be strictly correlated with histology. An observational retrospective study was conducted. Patients undergoing elective colectomy for uncomplicated diverticulitis between January 2014 and January 2016 in an academic medical center were collected. The majority of patients (46.2%) had previously encountered one episode of acute diverticulitis prior to colectomy, while 21.5% and 10.8% had experienced two and three or more prior episodes respectively. Most patients had recurrent or chronic abdominal pain in the left iliac fossa (66.2%) for diverticular disease and a large proportion also experienced constipation (40.0%). Diverticulitis was identified pathologically as being “mild” in 44.6% patients and “severe” in 55.4% patients. The mean age was significantly lower in patients with severe diverticulitis (56.7 years) than in patients with mild diverticulitis (67.0 years). 71.9% of males had severe diverticulitis compared to 39.4% of females. Males have a 3.9 times higher risk of histological severe diverticulitis than females (OR = 3.932; 1.390–11.122; p = 0.008). Multivariate logistic regression analysis confirmed that age and gender were independent factors associated with histological diagnosis. Single-institution data and retrospective design were main limitations of this study. Age and gender are independent factors associated with severity inflammation index derived at histological analysis and they could be translated to clinical practice to better categorize patients with uncomplicated diverticulitis at the bedside.

## Introduction

Diverticular disease is a condition in which small sacs (diverticula) form and bulge from the wall of the colon, causing constipation, cramps, bloating or bleeding. Diverticular disease is one of the most common diseases in Western countries to be diagnosed during colonoscopy^1^. Inflammation of the diverticula results in diverticulitis—a frequent complication of diverticular disease that affects ∼5–10% patients^2^. Diverticulitis can be classified as either uncomplicated or complicated, depending on the occurrence of an occlusion, perforation with secondary abscess, fistula and/or bleeding. The indications for elective colectomy in uncomplicated diverticulitis are a matter of debate yet, due to the fact that none of the recent guidelines supports such rules of the thumb^3,4^. As there is no strong scientific evidence regarding histology in diverticular disease, we retrospectively correlated the pathological features of uncomplicated diverticulitis surgical specimens with clinical data with the principal aim to compare the patient’s clinical picture with severity inflammation index of the colonic wall in order to find some predictive factors that may be strictly correlated with histology.

## Materials and methods

### Retrospective patient recruitment

Data from 65 consecutive patients with uncomplicated sigmoid diverticulitis according to the Hansen-Stock classification^5^, who underwent elective colectomy between January 2014 and January 2016, were analyzed retrospectively. The main surgical indications were the occurrence of recurrent or chronic abdominal pain in the left iliac fossa to refer exclusively to uncomplicated diverticulitis, uncomfortable constipation and/or failure to respond to common medical treatment (fibre-rich diet, mesalazine, rifaximine, probiotics), according to most international guidelines^4^. Ten parameters were selected and analyzed for each patient, based on: demographics (sex, age), clinical history (number of previous episodes of acute diverticulitis, recurrent or chronic abdominal pain, constipation), inflammatory index (white blood cell count [WBC], C-reactive protein [CRP] level) and radiological and/or endoscopic findings (thickening of the colonic wall and peri-visceral fat, sigmoid colon rigidity, narrowing of the intestinal lumen). Clinical parameters were analyzed through a careful patient’s clinical history evaluation and a complete interview with the patient. Bowel habits were evaluated by using the Gastrointestinal Symptom Rating Scale (GSRS), a questionnaire containing 15 questions on gastrointestinal symptoms^6^. Each question is scored according to a 7-points Likert scale, ranging from 1 (no discomfort at all) to 7 (very severe discomfort). Patients having scores ≥3 at visit 1 pre-operatively and at visit 2 at follow-up were defined as being ≪symptomatic≫. WBC and CRP measured levels were reported from the last pre-operative blood test, one week before colonic resection. The original diagnosis and the evidence that the symptoms mentioned were due to the presence of active but uncomplicated diverticulitis were confirmed by abdominal computed tomography (CT), which was performed one-week pre-operatively, with the evidence of left side diverticula, thickening of the colonic wall (≥5 mm) and involvement of pericolic fat, narrowing of the intestinal lumen, described in CT-reports. All radiological reports were performed by radiologists with a great expertise in colonic diseases. The sigmoid colon rigidity was defined by endoscopic reports before surgery. It was defined by the failure or impaired bowel distension at insufflations during colonoscopy examination. The main exclusion criterion was the presence of at least complication of diverticular disease in the patient’s history, including bleeding, abscess formation, evidence of extra-colonic gas, fistula formation, large bowel obstruction and free perforation with secondary peritonitis. All patients were followed every 6 months for a period of three years after surgery. Recurrence was defined by the presence of persistent left lower abominal pain, fever and leucocytosis after surgery.

The study was carried out in accordance with the International Ethical Guidelines and Declaration of Helsinki.

All patients signed a written informed consent before surgery. The aim of the study was only to review retrospective data from the clinical records of the patients. The authors Pesce A, La Greca G, Magro G, Latteri S and Puleo S had access to identifying patient information when analysing the data. Therefore, we requested a formal ethical approval for the retrospective data analysis and the use of patient samples, by obtaining an informed consent as part of the study, from the Institutional Review Board *“Comitato Etico Catania 1”* of University Hospital Policlinico-Vittorio Emanuele prior to carrying the study out. The requested approval was granted. Particular emphasis has been placed on the confidential aspects of data and the anonymity of patients in scientific research; this issue is of great interest to the Privacy Authority (General authorization to process personal data for scientific research purposes. Official Journal of the Republic of Italy, No. 72, 26 March 2012).

### Histological analysis

**Figure 1:**
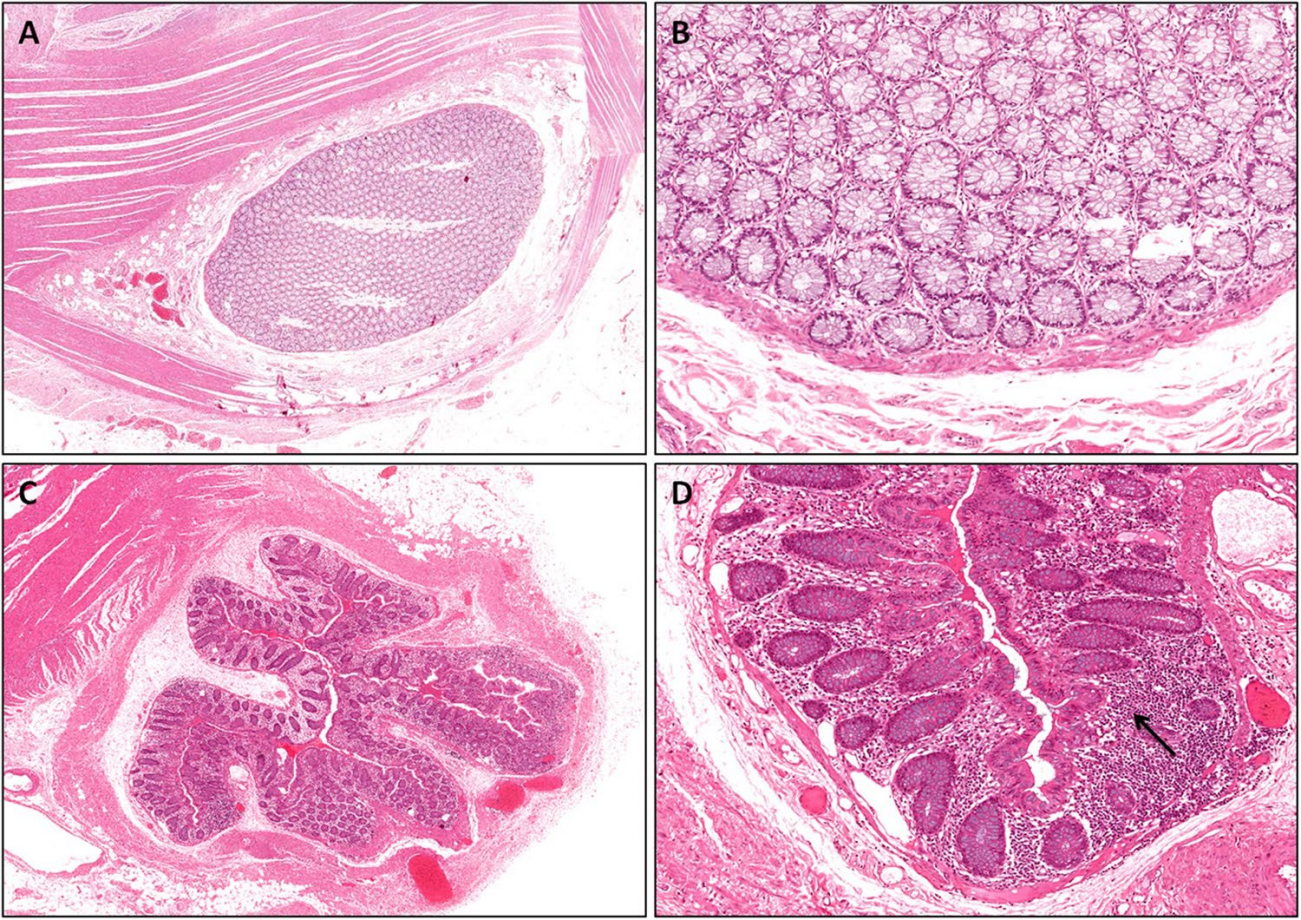
Histological analysis of surgical sections from a patient with mild diverticulitis. (**A**,**B**) Grade I diverticulitis: hematoxylin and eosin (H&E) staining of normal-appearing mucosa at low (**A**) and high magnification (**B**). (**C D**) Grade II diverticulitis: H&E staining showing moderate-to-severe inflammation restricted to the mucosa at low (**C**) and high magnification (**D**, black arrow).

**Figure 2:**
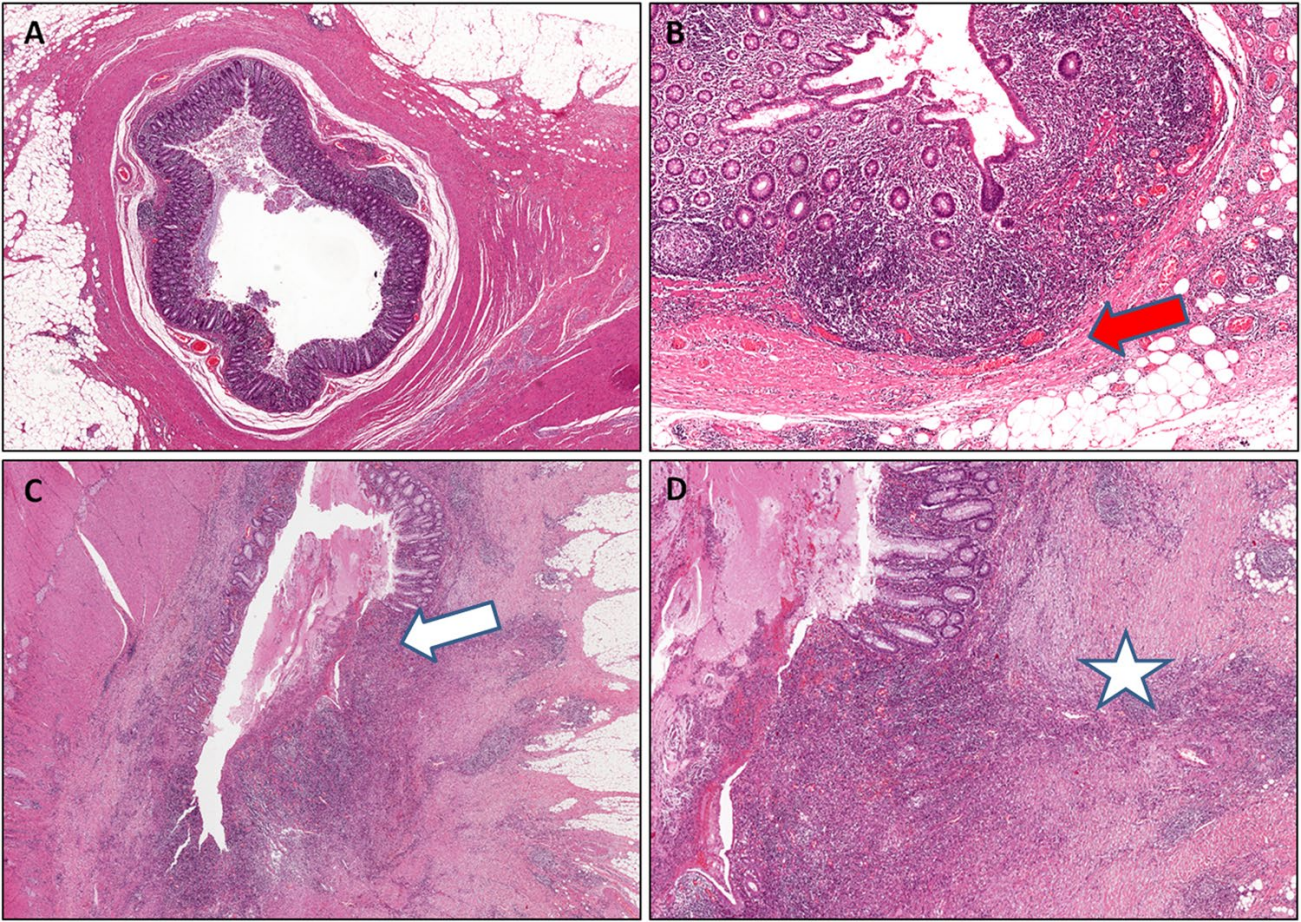
Histological analysis of surgical sections from a patient with severe diverticulitis. (**A**,**B**) Grade III diverticulitis: hematoxylin and eosin (H&E) staining showing moderate-to-severe inflammation involving the smooth muscle layer with focal extension to the sub-serosa, at low (A) and high magnification (**B**, red arrow). (**C**,**D**) Grade IV diverticulitis: H&E staining showing ulceration of the mucosa (white arrow) and diffuse inflammation involving the subserosa with reactive fibrous tissue (red star), at low (**C**) and high magnification (**D**).

As no histological classification is available, histological examination of the surgical specimens was performed following a severity index grading scale which was arbitrarily graded as follows: (a) Grade I: diverticula without notable evidence of inflammation (Fig. 1A,B); (b) Grade II: diverticulitis restricted to the mucosa (with moderate-to-severe inflammation, variably associated with cryptitis and/or crypt abscesses in the lamina propria that exhibit either normal or distorted glands) with focal extension (Fig. 1C,D); (c) Grade III: diverticulitis as for Grade II that extends to the smooth muscle layer, with focal and limited inflammation extended to the subserosa (Fig. 2A,B); (d) Grade IV: diverticulitis with diffuse involvement of the subserosa, with potential risk of perforation (Fig. 2C,D). Grades I-II were considered “mild diverticulitis”, whereas Grades III-IV were considered “severe diverticulitis”. Hematoxylin and Eosin (H&E) staining was performed to assess the histology.

All elective colectomies (in particular 60 sigmoidectomies and 5 left colectomies) were performed by the same team of surgeons, regardless of laparoscopic (n°25) or open approach (n°40). The surgical specimens were evaluated by one pathologist with expertise in gastro-intestinal diseases, blinded to the clinical condition and health of the patient. In our anatomic pathology laboratory surgical pathologists are recommended to sample as many diverticula as possible. As there is no international protocol, the number of blocks containing the surgical specimen range from a minimum of 8 to a maximum number of 16. The final number for each patient was closely related to the extension of the surgical specimen and the number of grossly identified diverticula. In the cases with numerous diverticula, surgical pathologists sampled those, which were mainly suspected for inflammation.

### Statistical analyses

The SPSS software (version 22.0, SPSS, Chicago, IL) was used for all statistical analyses. Descriptive statistics including frequencies, means ± standard deviations (SDs), median values and range were used to characterize the study population. Patients with mild diverticulitis were compared with patients with severe diverticulitis: frequencies were compared by two-tailed Chi-squared test and quantitative variables by Student’s t test. Odds ratios (ORs) and the corresponding 95% confidence intervals (95% CIs) were computed to measure the association level. Unconditional multivariable logistic regression analysis was used to adjust for age (using the median value as cut off) and gender. The stated hypothesis was to assess whether eligible demographics, clinical, serologic and radiological factors may be predictive of severe histology in uncomplicated diverticulitis. The adjusted ORs with the respective 95% CIs are reported. A p-value <0.05 was considered statistically significant in all analyses.

## Results

During the study period, 65 patients (mean age 61.4 ± 13.4 years) were identified with uncomplicated diverticulitis who underwent elective colectomy. The main characteristics of the study population are reported in Table 1 in terms of demographic factors, clinical history and histologic presentation. The majority of patients (46.2%) had previously encountered one episode of acute diverticulitis prior to colectomy while 21.5% and 10.8% had experienced two and three or more prior episodes respectively. This group of patients with one single episode of acute diverticulitis complained recurrent or chronic abdominal pain and failure to respond to medical treatment with consequent indication for surgery. Most patients had recurrent or chronic abdominal pain in the left iliac fossa (66.2%) for diverticular disease and a large proportion also experienced constipation (40.0%). As regards to medical treatments, fibre-rich diet was used in all patients; rifaximine was used in 69.2% of patients, mesalazine and probiotics in the remnant 30.8%. At the radiologic level, most patients exhibited thickening of the colonic wall and peri-visceral fat (66.2%). In sum, most patients were categorized histologically into inflammation grades II (30.8%) or III (38.5%), and 44.6% and 55.4% patients were considered to exhibit “mild diverticulitis” and “severe diverticulitis”, respectively (Table 1).

**Table 1:** Patient demographics, clinical history and histologic presentation.

Study cohort descriptors	N (%)
***Patients (n*** **=** ***65):***	
Male	32 (49.2)
Female	33 (50.8)
*Laboratory tests:*	
CRP ≥ 10 ng/mL	29 (44.6)
CRP < 10 ng/mL	36 (55.4)
WBC count ≥12.400/µL	11 (16.9)
WBC count <12.400/µL	54 (83.1)
***History of acute diverticulitis:***	
None	8 (12.3)
1 episode	30 (46.2)
2 episodes	14 (21.5)
3 episodes	6 (9.2)
>3 episodes	7 (10.8)
***Clinical symptoms:***	
Recurrent or chronic abdominal pain in left iliac fossa	43 (66.2)
Constipation	26 (40.0)
***Radiologic findings:***	
Thickening of the colonic wall and peri-visceral fat	43 (66.2)
Sigmoid colon rigidity	9 (13.8)
Narrowing of the intestinal lumen	23 (35.4)
***Inflammation grading:***	
Grade I: diverticula without any significant sign of inflammation	9 (13.8)
Grade II: diverticulitis restricted to mucosa with focal extension	20 (30.8)
Grade III: diverticulitis with extension to smooth muscle layer	25 (38.5)
Grade IV: diverticulitis with diffuse subserosa involvement	11 (16.9)
***Histologic grading:***	
“Mild” diverticulitis	29 (44.6)
“Severe” diverticulitis	36 (55.4)

Abbreviations: CRP, C-Reactive Protein; WBC, White Blood Cells.

The comparison of characteristics between patients with mild diverticulitis and patients with severe diverticulitis is show in Table 2. Patients with severe diverticulitis were significantly younger than those with mild diverticulitis (56.7 vs. 67.0 years, respectively; p = 0.002). This finding remained valid when considering the median age (64 years) as the cut-off value (p = 0.001). More males than females had severe diverticulitis (71.9 vs. 39.4%, respectively), resulting in a 3.9 times higher risk (OR: 3.932; 95%CI: 1.390–11.122). There was no statistically significant difference in the average number of previous episodes of acute diverticulitis between those with severe and mild diverticulitis (Table 2). Using multivariate logistic regression analysis, we confirmed that age and gender are independently associated with histological diagnosis of diverticulitis. In particular, we found that patients >64 years (OR: 0.156; CI95%: 0.048–0.509; p = 0.002), or females (OR: 0.272; CI95%: 0.083–0.892; p = 0.032) have a reduced risk of severe diverticulitis, based on histologic examination.

**Table 2:** Patient demographics and clinical history according to mild or severe diverticulitis.

Study cohort descriptors	Mild diverticulitis	Severe diverticulitis	Univariate analysis p-value (OR; CI95%)	Multivariate analysis p-value (OR; CI95%)
Age. mean (years)	67 (n = 30)	56.7 (n = 35)	p = 0.002	
Median age (64 ys)	>64 ys: 67.9% (n = 19)	>64 ys: 24.2% (n = 8)	p = 0.001 (0.152; 0.049–0.466)	p = 0,002 (0,156; 0,048–0,509)
Gender				
Male	28.1% (n = 9)	71.9% (n = 23)	p = 0.008	p = 0,032
Female	60.6% (n = 20)	39.4% (n = 13)	(3.932; 1.390–11.122)	(0,272; 0,083–0,892)
Mean no. of previous acute diverticulitis episodes	1.5 (n = 29)	1.7 (n = 36)	p > 0.05	NS
C-reactive protein				
≥10 ng/mL	46.7% (n = 7)	42.1% (n = 8)	p = 0.790	NS
White blood cells				
≥12.400/µL	17.2% (n = 5)	16.7% (n = 6)	p = 0.951	NS
Abdominal pain (yes)	65.5% (n = 19)	69.4% (n = 25)	p = 0.736	NS
Constipation (yes)	34.5% (n = 10)	44.4% (n = 16)	p = 0.415	NS
Thickening of the colonic wall and peri-visceral fat (yes)	58.6% (n = 17)	72.2% (n = 26)	p = 0.249	NS
Sigmoid colon rigidity (yes)	13.8% (n = 4)	13.9% (n = 5)	p = 0.991	NS
Narrowing of intestinal lumen (yes)	41.4% (n = 12)	30.6% (n = 11)	p = 0.364	NS

Abbreviations: *OR: Odds Ratio; CI95%: Confidence Interval 95%; NS: not significant*.

During the follow-up period, 92.3% of patients solved their problems after colectomy, with 7.7% reporting bloating and frequent bowel movements defined after a careful interview with the patient by applying the Gastrointestinal Symptom Rating Scale (GSRS) score. Recurrence rate was reported in 5 patients (7,7%) during the follow-up period, for the record 2 patients at 6 months and three patients at two years after surgery. All these patients were treated conservatively.

## Discussion

There are numerous practice guidelines for the diagnosis and treatment of diverticular disease. Indeed, a recent systematic review of national and international guidelines identified that eleven different guidelines have been compiled over the past ten years alone^4^. Despite this wealth of guidance, there is no general consensus regarding the treatment of uncomplicated diverticulitis. According to these 11 guidelines, the clinical decision mainly depends on individual patient-related factors, such as co-morbidities or the impact of symptoms on quality of life. In this regard, the presence of recurrent or chronic abdominal pain related to uncomplicated diverticulitis and uncomfortable constipation, are generally the most pronounced clinical symptoms to affect quality of life. Age also has an important role in clinical decision-making: young patients should be operated due to their expected longer life expectancy with opportunity for multiple episodes of diverticulitis^4^. Finally, guidance supports that prophylactic elective colectomy should be preferred when conservative therapy fails. Most patients experience different medical treatments during the clinical course of the diverticular disease but in some cases they prove to be ineffective.

To the best of our knowledge, this study is the first to investigate a possible correlation between the severity of inflammation of the colonic wall and the patient’s clinical picture in uncomplicated diverticulitis. This study was conceived from evidence at our institution showing that the degree of inflammation identified in pathological reports was often discordant with the clinical signs of patients with uncomplicated diverticulitis and who had undergone elective colonic resection. Accordingly, we retrospectively analyzed a series of 65 patients with histologically proven uncomplicated diverticulitis to correlate histological findings with patient clinical history. The primary outcome was to find some predictive factors correlated with histology in uncomplicated diverticular disease. We found that age and gender are independent risk factors for severe inflammation that is detectable at histologic diagnosis. Patients greater than 64 years and females of any age have a lower chance of the pathologist finding severe inflammation, after the decision for surgery, and surgery itself, have been performed. Probably, young patients have a reduced compliance to medical treatments and they could have a more marked inflammatory activity in comparison to elderly patients. Although a certain predisposition by genders for the diverticular disease cannot be found, some studies suggest that hormones may play a role^7^. We think that these results may provide an important consideration to the clinical work-up of diverticular disease.

Moreover, there is no strong scientific evidence regarding histology in diverticular disease. In 2008, Tursi A *et al*.^8^ assessed and graded the mucosal inflammatory infiltrate in different degrees of diverticular disease and they compared them with healthy controls. The authors found an increased inflammatory infiltrate in diverticular disease according to the different degree of the disease (asymptomatic diverticulosis, symptomatic uncomplicated diverticular disease, and acute uncomplicated diverticulitis) and higher than healthy controls. Moreover, also asymptomatic diverticulosis showed higher inflammatory cell density than controls. In 2010, the same authors analyzed the predictive value of serological markers, such as white blood cells, erytro-sedimentation rate, C-reactive protein (CRP) with histology in acute uncomplicated colonic diverticulitis^9^. They found a direct relation between the serological markers and the histologic damage. Moreover, CRP was found to be the most sensitive marker of mild-moderate histologic damage. In our study WBC and CRP levels were not associated to histology severity in uncomplicated diverticulitis.

In another study by Tursi A *et al*.^10^ the detection of endoscopic and histological inflammation signs after a first attack of acute uncomplicated diverticulitis was found to be a predictive factor of recurrence in acute diverticulitis, a behaviour very similar to inflammatory bowel diseases.

However, neurotransmitter imbalance has been hypothesised as a pathogenetic mechanism in several bowel conditions, even in uncomplicated diverticular disease. Jeyarajah S *et al*.^11^ performed a clinicopathological study of serotonin of sigmoid colon mucosa in uncomplicated diverticulitis and they found that serotonin expression is not involved in the pathogenesis of chronic symptoms in diverticular disease.

In the current study, we also found that a significant number of patients (44.6%) who underwent elective surgery had only pathologically mild diverticulitis, by highlighting the discrepancy between clinical and histological findings. In the absence of well-established guidelines, the potential for any surgical complication (such as post-operative morbidity or death) in patients with histologically proven mild diverticulitis could represent the basis for a medical negligence claim. In clinical practice, conservative treatment, outside of surgery, represents the first choice for patients with uncomplicated diverticulitis, but there are divergent recommendations^4^. Moreover, some studies reported that recurrence rate of multiple episodes of diverticulitis is 19–54% at 5 year follow-up^12^. At the same time, elective surgery can be considered the definitive treatment as it removes definitively the cause of clinical discomfort for patients, by improving in most cases the quality of life expectancy. Recurrence rates after appropriate sigmoid resection ranges from 5% to 11% and some patients need urgent reoperation^13–15^. Recurrence is usually defined by the presence of persistent left lower abominal pain, fever and leucocytosis in a variable time after surgery. Andeweg C *et al*.^14^ demonstrated that younger age and the persistence of post-operative symptoms predict a recurrence of diverticulitis after resection. In our study recurrence rate was reported in 5 patients (7,7%), in agreement with the data from the literature.

We would recommend that in cases of symptomatic uncomplicated diverticulitis, the decision for elective surgery should be made on a case-by-case basis, which has been the standard approach for many years^15–17^. Physicians should ensure that patients are aware of the specific risks and benefits, based on current scientific evidence^18–22^. Accordingly, the decision to perform elective prophylactic colonic resection should be individualized^1,19,23–25^ and always conducted under best clinical practice.

### Limitations

This study presents some limitations related to a retrospective analysis and the current data require further elaboration in studies on large numbers of patients undergoing elective colectomy for uncomplicated diverticulitis in order to implement the study results to the existing guidelines. Another limitation of the study is represented by the possible selection of other clinical parameters, which could correlate with pathological findings.

## Conclusions

Age and gender are independent factors associated with severity inflammation index derived at histological analysis and they could be translated to clinical practice to better categorize patients with uncomplicated diverticulitis at the bedside.

Part of these data has been presented at the following meeting: Italian Society of Surgical Researches (SIRC) Meeting, Rome 14–18 October 2018.
